# Characteristics of intermediate age-related macular degeneration with hyperreflective foci

**DOI:** 10.1038/s41598-022-23380-w

**Published:** 2022-11-01

**Authors:** Wataru Kikushima, Yoichi Sakurada, Atsushi Sugiyama, Seigo Yoneyama, Mio Matsubara, Yoshiko Fukuda, Taiyo Shijo, Yumi Kotoda, Serena Fragiotta, Kenji Kashiwagi

**Affiliations:** 1 Department of Ophthalmology, University of Yamanashi, Shimokato 1110, Chuo, Yamanashi 409-3898 Japan; 2 Ophthalmology Unit, Department NESMOS, S. Andrea Hospital, University of Rome “La Sapienza”, 00189 Rome, Italy

**Keywords:** Eye diseases, Macular degeneration

## Abstract

Hyperreflective foci (HRF) are the findings observed in optical coherence tomography (OCT) in several retinal diseases and are believed to be associated with the increased risk of atrophy in eyes with age-related macular degeneration (AMD). In this study, we investigated the clinical and genetic characteristics of intermediate AMD with HRF. We reviewed the medical charts for 155 patients with intermediate AMD, in whom macular neovascularization (MNV) was observed in the contralateral eye. The presence or absence of an HRF was evaluated using a spectral-domain OCT volume scan spanning the macular region. Patients were followed longitudinally for at least 12 months, and the maximum follow-up period was 60 months. Genotyping of *ARMS2* A69S and *CFH* I62V was performed in all participants. Of the 155 patients (mean age: 77.8 ± 7.6 years, male/female: 103/52), HRF was observed in 53 eyes (34.2%) and was significantly associated with type-3 MNV (*p* = 1.0 × 10^−5^) in the contralateral eye, pseudodrusen (*p* = 5.0 × 10^−4^), thinner subfoveal choroidal thickness (*p* = 0.013), and risk of *ARMS2* A69S (*p* = 0.023). During follow-up (40.8 ± 17.5), 38 eyes (24.5%) developed advanced AMD. The mean time to the onset of advanced AMD was 29.8 ± 12.9 months in eyes with intermediate AMD. HRF was associated with MNV (*p* = 1.0 × 10^−3^), but not with atrophy.

## Introduction

Drusen generally accumulate beneath the retinal pigment epithelium (RPE) and are hallmarks of age-related macular degeneration (AMD). As per the Beckman Initiative for Macular Research Classification Committee, drusen are categorized into small (< 63 µm), medium (63 µm ≤ and ≤ 125 µm), and large (> 125 µm) drusen^1^. In this classification, intermediate AMD is defined as eyes with large drusen or with pigmentary abnormalities associated with at least medium druse. On another front, the AREDS classification system is a widely used classification system for AMD^1^. One of the major differences between the two classification systems is that the AREDS classification system includes small drusen as Level 1 AMD, whereas the Beckman Initiative regards small drusen without AMD pigmentary abnormalities as the normal aging change. In addition, the AREDS classification system evaluates the total area of the drusen, which is not included in the Beckman Initiative system.

Hyperreflective foci (HRF) are visualized at any retinal layer through spectral-domain/swept-source optical coherence tomography (SD/SS-OCT) in several retinal diseases and are defined as well-demarcated lesions with equal reflectivity to the RPE bands. They are sometimes detected as hyperpigmentation on color fundus photography^2^. They are believed to migrate toward the vitreous side of the retina from the RPE in eyes with early/intermediate AMD. Drusen under the HRF are more likely to collapse and develop atrophy. Several studies have demonstrated that the number and volume of HRF are associated with an increased risk of atrophy, but not macular neovascularization (MNV) in Caucasians^3,4^.

The risk of AMD varies depending on the drusen type and size, and RPE abnormalities. The contralateral eye is at a high risk of developing advanced AMD. A randomized clinical trial suggested that the risk of progression from intermediate to advanced AMD increased if the patient had already developed advanced AMD in the fellow eye^5^.

In this study, we compared the genetic and clinical characteristics of intermediate AMD with or without HRF, and longitudinally evaluated the incidence of advanced AMD using unilateral MNV cases.

## Results

**Figure 1:**
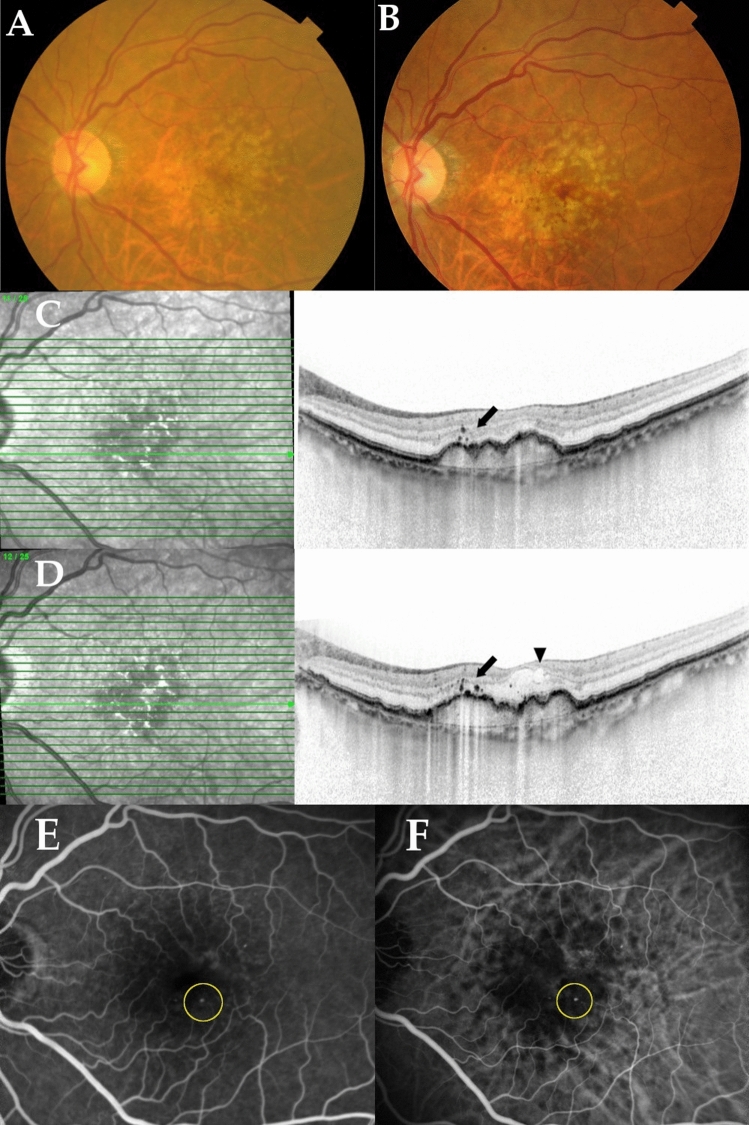
A representative case of a 71-year-old woman with intermediate age-related macular degeneration (AMD) colocalizing with hyperreflective foci (HRF) in the left eye and type-3 macular neovascularization (MNV) in right eye. (**A**) Color fundus photography of the left eye at the initial appearance demonstrates drusenoid pigment epithelial detachment (DPED) surrounded by reticular pseudodrusen in the macular area. (**B**) At 7 months after initial presentation, color fundus photography of the left eye demonstrates the development of intraretinal hemorrhage in the macular area. (**C**) A horizontal scan through the fovea by spectral-domain optical coherence tomography (SD-OCT) in the left eye at the initial appearance shows HRF (black arrow) near the protrusion of DPED. (**D**) A horizontal SD-OCT scan through the fovea of the left eye at 7 months shows the presence of intraretinal fluid (black arrowhead) and increased HRF (black arrow). (**E**) Fluorescein angiography and (**F**) indocyanine angiography of the left eye at 7 months shows a hot spot (yellow circle) that is consistent with type-3 MNV.

A total of 155 patients with intermediate AMD, who had treatment naïve MNV in the contralateral eye at baseline, were included in this study. Table 1 shows the baseline demographic and genetic characteristics of participants. The mean age was 77.8 ± 7.6 years and 52 patients (33.5%) were women. Of 155 eyes, HRF was observed in 53 eyes (34.2%). The MNV subtypes in the contralateral eye were type 3 MNV (n = 23, 14.8%), typical neovascular AMD (n = 82, 52.9%), and polypoidal choroidal vasculopathy (PCV, n = 50, 32.3%). Figure 1 shows representative cases of HRF at initial presentation that indicate the development of type 3 MNV during the follow-up period.

**Table1:** Baseline demographic and genetic characteristics.

Age(years)	77.8 ± 7.6
Female/male	52/103
Presence of hyperreflective foci	53 (34.2%)
Presence of DPED	43 (27.7%)
Presence of pseudodrusen	64 (41.3%)
Subfoveal choroidal thickness (µm)	213 ± 89
**MNV subtype in the contralateral eye**	
Type 3 MNV (RAP)	23 (14.8%)
Typical neovascular AMD	82 (52.9%)
PCV	50 (32.3%)
***ARMS2* A69S (rs10490924)**	
TT	88 (56.8%)
TG	51 (32.9%)
GG	16 (10.3%)
***CFH* I62V (rs800292)**	
GG	93 (60.0%)
GA	52 (33.5%)
AA	10 (6.5%)

MNV, macular neovascularization; DPED, drusenoid pigment epithelial detachment; RAP, retinal angiomatous proliferation; AMD, age-related macular degeneration; PCV, polypoidal choroidal vasculopathy.

Table 2 shows the characteristics of the patients with and without HRF. The proportion of pseudodrusen (60.4%) and drusenoid pigment epithelial detachment (54.7%) was significantly higher in the HRF (+) group than that in HRF (−) group (*p* = 5.0 × 10^−4^ and *p* = 6.4 × 10^−8^, respectively). In addition, patients in the HRF (+) group had thinner subfoveal choroidal thickness (*p* = 0.013), a higher proportion of type 3 MNV (*p* = 1.0 × 10^−5^), lower proportion of PCV in the contralateral eye (*p* = 9.8 × 10^−4^), and higher risk allele frequency of *ARMS2* A69S (*p* = 0.023) than those in HRF (−) group.

**Table 2:** Comparison between eyes with and without hyperreflective foci.

	HRF (+) (n = 53)	HRF (−) (n = 102)	*p*-value
Age(years)	78.8 ± 7.6	77.3 ± 7.6	0.22
Females	14 (26.4%)	38 (37.3%)	0.18
Presence of pseudodrusen	32 (60.4%)	32 (31.4%)	5.0 × 10^−4^
Presence of DPED	29 (54.7%)	14 (13.7%)	6.4 × 10^−8^
Subfoveal choroidal thickness	189 ± 86	226 ± 88	0.013
**MNV subtype in the contralateral eye**			
Type 3 MNV	17 (32.1%)	6 (5.9%)	1.0 × 10^−5^
Typical neovascular AMD	28 (52.8%)	54 (52.9%)	0.99
PCV	8 (15.1%)	42 (41.2%)	9.8 × 10^−4^
T allele frequency of ARMS2 A69S	81.1%	69.1%	0.023
G allele frequency of CFH I62Vj	78.3%	76.0%	0.65

HRF, hyperreflective foci; DPED, drusenoid pigment epithelial detachment; MNV, macular neovascularization; RAP, retinal angiomatous proliferation.

### Progression from intermediate to advanced AMD

The mean follow-up period was 40.8 ± 17.5 months. During the follow-up period, 38 eyes (24.5%) progressed from intermediate to advanced AMD in the study eye. The mean duration from the first visit to the onset of advanced AMD was 29.8 ± 12.9 months. The mean follow-up period was significantly shorter in the eyes that developed advanced AMD than that in the eyes without developing advanced AMD (29.8 ± 12.9 months vs. 44.4 ± 17.4 months, *p* = 5.0 × 10^−6^). Of the 38 eyes with advanced AMD, 16 showed type-3 MNV, 10 showed typical neovascular AMD, 4 showed PCV, and 9 showed atrophy. One eye showed simultaneous progression to type-3 MNV and atrophy.

During the follow-up period, 23 (43.4%) eyes developed advanced AMD, including 20 MNV eyes (one type 1 MNV, 8 type 2 MNV, and 11 type 3 MNV) and 3 atrophy eyes in the HRF (+) group; and 15 (14.7%) eyes developed advanced AMD, including 9 MNV eyes (three type 1 MNV, 2 type 2 MNV, and 4 type 3 MNV), 4 atrophy eyes, and one eye with simultaneous type 3 MNV and atrophy in the HRF (−) group. The subtypes of newly-developed MNV were same as those of the contralateral eyes in 17 (85.0%) eyes in the HRF (+) group and 4 (40%) eyes in the HRF (−) group. Among 23 eyes that developed from intermediate to advanced AMD in the HRF (+) group, 18 (78.3%) eyes also showed HRF in the contralateral eye with treatment naïve MNV at baseline. However, it is difficult to differentiate accurately between HRF with RPE migration and HRF derived from lipids in exudative changes by SD/SS-OCT only. In the HRF (−) group, four (3.9%) eyes developed HRF during the study period; however, no eyes progressed to advanced AMD. During the follow-up period, no eye showed the decrease or disappearance of HRF in the HRF (+) group.

In order to investigate the predictor of developing from intermediate to advanced AMD, we conducted Cox regression analysis. As a result, the baseline best-corrected visual acuity (BCVA), presence of pseudodrusen, and presence of HRF were associated with advanced AMD (Table 3). Cox regression analyses were performed to investigate the risk of MNV (Table 4) and atrophy (Table 5) in a similar fashion. The analyses suggested that the presence of HRF at baseline was associated with MNV (*p* = 0.001, hazard ratio: 3.67, 95% CI 1.68–8.00), and the presence of pseudodrusen (*p* = 0.02, hazard ratio: 11.65, 95% CI 1.44–94.03) was associated with progression to atrophy.

**Table 3:** Cox regression analyses associated with advanced AMD (repeated forward selection method).

Variables	β-coefficient	*p*-value	Hazard ratio	95% CI
Baseline BCVA	2.38	0.01	10.78	1.72–67.6
Pseudodrusen	0.90	0.013	2.47	1.21–5.06
Hyperreflective foci	0.70	0.049	2.01	1.00–4.03

Age, sex, drusenoid pigment epithelial detachment, baseline subfoveal choroidal thickness, *ARMS2* A69S, and *CFH* I62V were eliminated in this analysis.

AMD, age-related macular degeneration; BCVA, best-corrected visual acuity.

**Table 4:** Cox regression analyses associated with macular neovascularization (repeated forward selection method).

Variables	β-coefficient	*p*-value	Hazard ratio	95% CI
Baseline BCVA	1.64	0.10	5.17	0.73–36.8
Hyperreflective foci	1.30	0.001	3.67	1.68–8.00

Age, sex, drusenoid pigment epithelial detachment, pseudodrusen, baseline subfoveal choroidal thickness, *ARMS2* A69S, and *CFH* I62V were eliminated in this analysis.

BCVA, best-corrected visual acuity.

**Table 5:** Cox regression analyses associated with atrophy (repeated forward selection method).

Variables	β-coefficient	*p*-value	Hazard ratio	95% CI
Baseline BCVA	3.68	0.07	39.64	0.75–2094
Pseudodrusen	2.46	0.02	11.65	1.44–94.03

Age, Sex, drusenoid pigment epithelial detachment, hyperreflective foci, baseline subfoveal choroidal thickness, *ARMS2* A69S, and *CFH* I62V were eliminated in this analysis.

BCVA, best-corrected visual acuity.

### Kaplan–Meier survival analysis

**Figure 2:**
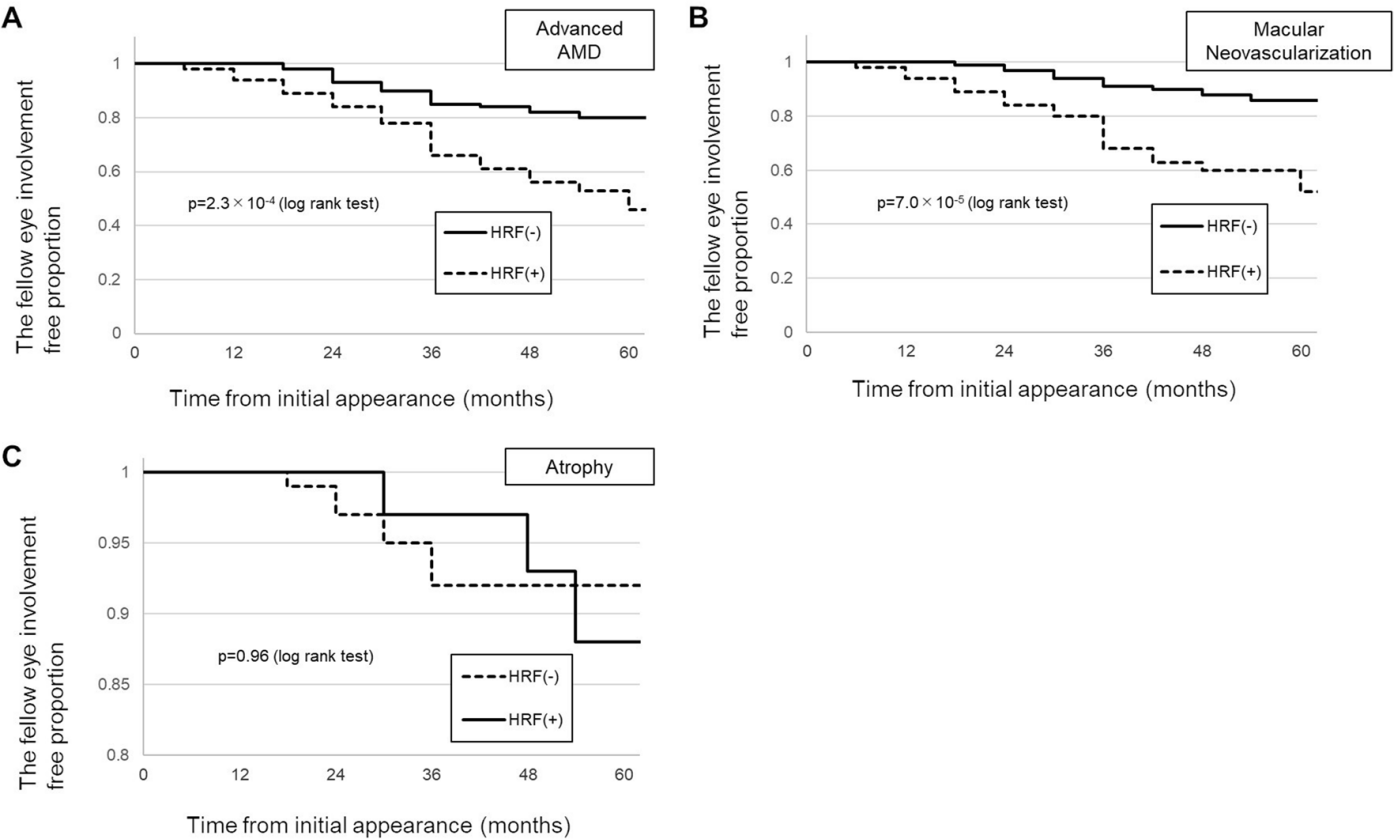
Kaplan–Meier survival curve demonstrating progression from intermediate to advanced AMD with or without HRF. (**A**) Kaplan–Meier survival curve (KM curve) of the time to progression to advanced AMD. The proportion of patients who progressed to advanced AMD at 60 months is 53.8% and 19.6% in the HRF (+) and HRF (−) groups, respectively. HRF is associated with the progression and time to progression of advanced AMD (*p* = 2.3 × 10^−4^, log-rank test). (**B**) KM curve of the time to progression to MNV. The proportion of patients who progressed to MNV at 60 months is 47.5% and 14.2% in the HRF (+) and HRF (−) groups, respectively. HRF is associated with the progression and time to progression of MNV (*p* = 7.0 × 10^−5^, log-rank test). (**C**) KM curve of the time to progression to atrophy. The proportion of patients who progressed to atrophy at 60 months is 11.8% and 7.6% in the HRF (+) and HRF (−) groups, respectively. There are no significant differences between the presence of HRF and time to progression of atrophy (*p* = 0.96, log-rank test).

Figure 2 illustrates the Kaplan–Meier survival curve for the involvement-free proportion, which means the proportion of the eyes without developing from intermediate to advanced AMD, MNV and atrophy of the fellow eye. In the HRF (+) group, 20 patients (37.7%) progressed to MNV from intermediate AMD at 60 months from initial appearance, and the proportion was significantly higher than that in HRF (−) group (9.8%, *p* = 7.0 × 10^−5^, log-rank test). In contrast, the proportion of patients with progression to atrophy between the HRF (+) and HRF (−) groups was not statistically significant (5.7% vs. 5.9%, *p* = 0.96, log-rank test).

## Discussion

The present study attempted to characterize intermediate AMD with HRF in 155 patients with MNV versus those without HRF. Several studies have investigated OCT biomarkers as predictors of conversion from intermediate to advanced AMD and have reported that the risk of progression varies depending on the drusen type, shape, reflectivity, and internal homogeneity. In addition to drusen substructures^4^, HRF is considered as another OCT biomarker for advanced AMD.

In eyes with intermediate AMD, HRF may represent RPE migration because RPE has the potential to migrate when induced by cytokines and inflammatory factors in response to oxidative stress and complement activation^6,7^.

In Caucasian people, HRF was observed in 41–50% of the eyes with intermediate AMD, that is higher in this study (32%)^4,8,9^. However, HRF was observed in 74% (17/23) of the contralateral eyes with type-3 MNV and 50% (32/64) of eyes with pseudodrusen. HRF may be prevalent due to differences in the prevalence of these entities.

Tiosano et al. recently evaluated choriocapillaris flow deficits (CC-FD) in eyes with HRF using OCT angiography. The study demonstrated that CC-FD was greater in the eyes with HRF than in those without HRF, based on 55 eyes with intermediate AMD^10^. Corvi et al. reported that a greater CC-FD was associated with progression to complete RPE and outer retinal atrophy (cRORA) in the eyes with intermediate AMD^11^. Several groups have demonstrated that flow signals decreased at the choriocapillaris level, and that CC-FD areas increased in the eyes with pseudodrusen^12,13^. In the present study, both HRF and pseudodrusen increased the risk of advanced AMD. As these entities are colocalized, advanced AMD may develop through a similar pathway.

Several studies have longitudinally evaluated intermediate AMD. The AREDS2 Ancillary SD-OCT Study Group reported that the presence of HRF, and number and distribution of HRF are associated with an increased risk of atrophy at 2 years in eyes with intermediate AMD^4^. Lei et al. evaluated the progression of AMD using OCT-based scoring system and reported that the presence of HRF is a risk factor for atrophy or cRORA^14^. Nassisi et al. quantified HRF volume in the eyes with intermediate AMD and concluded that the area of HRF correlated with a 1-year risk of progression to atrophy, but not MNV^3^. These studies were exclusively performed on Caucasian people and demonstrated strong association of HRF with atrophy rather than with MNV. In the present study, HRF was strongly associated with MNV but not with atrophy. This result is in contrast to those of previous studies. There are several possible explanations for this finding. First, the study eye was the contralateral eye with MNV; therefore, the study eye was more likely to develop MNV than cRORA. In the previous study aforementioned, Christenbury et al. included only one eye with category 3 AMD (several medium drusen or at least one large druse) and excluded eyes with category 4 AMD (central geographic atrophy or choroidal neovascularization). Lei et al. included the patients with early AMD in one eye with or without late AMD in the fellow eye. In addition, they included not only HRF and RPD but also drusen volume and hyporeflective foci within the drusenoid lesion as risk factors. Nassisi et al. included the patients with intermediate AMD in one eye or both eyes and did not include the patients with advanced AMD in the contralateral eye. Furthermore, they assessed HRF not only qualitatively but quantitatively. These differences in the study design might have affected the results. Second, geographic atrophy/cRORA is a rare phenotype in advanced AMD in Asians than in Caucasian people^15^; thus, the present result may be due to differences in ethnicities.

In terms of genetic association, only one study performed a genetic analysis of HRF. In a previous study, risk variants *ARMS2* (rs104909) and *CFH* I62V (rs800292) were associated with an increased risk of HRF with hazard ratio of 1.64 and 1.49, respectively, in the eyes with intermediate AMD^16^. In the present study, the hazard ratios of *ARMS2* (rs10490924) and *CFH* I62V were 1.82 and 1.14, respectively. Based on results of previous and present studies, the risk variants of *ARMS2*, than of *CFH*, may be more strongly associated with HRF.

The merits of the present study are the long-term follow-up of a large number of participants. However, the present study has some limitations. First, the study was retrospective. Second, we failed to perform quantitative HRF evaluation. The information on changes in the number or distribution of HRF and the blood flow evaluation around drusen or HRF may provide some insights into the progression of advanced AMD^17^.

In summary, HRF was more frequently observed in type-3 MNV and was associated with pseudodrusen, drusenoid PED, thinner subfoveal choroidal thickness, risk variants of *ARMS2*, and progression to MNV.

## Methods

### Participants

We retrospectively reviewed the medical charts of patients with intermediate AMD in whom MNV was observed in the contralateral eye and who visited the Macular Clinic of the Yamanashi University Hospital between March 2015 and March 2021. This retrospective study was approved by the Institutional Review Board of the University of Yamanashi and conducted in accordance with the principles of the Declaration of Helsinki. Written informed consent was obtained from all the participants before enrollment to the study.

At the initial presentation, all patients underwent comprehensive ophthalmic examination, including measurement of BCVA using a Landolt chart and intraocular pressure, slit-lamp biomicroscopy with or without a 78 diopter lens with pupil dilation, fundus photography, fluorescein/indocyanine green angiography (FA/ICGA) using a confocal scanning laser ophthalmoscope (HRA2, Spectralis, Dossemheim, Germany), fundus autofluorescence, and near-infrared reflectance and SD-OCT using Spectralis.

The inclusion criteria were as follows: (1) fellow eyes with intermediate AMD in patients with unilateral treatment—naive MNV, including typical neovascular AMD, polypoidal choroidal vasculopathy (PCV), and retinal angiomatous proliferation, also known as type-3 MNV; and (2) a minimum follow-up period of 12 months after the initial presentation. Intermediate AMD was diagnosed according to previously reported criteria^1^.

The exclusion criteria were as follows: (1) previous vitrectomy and (2) other ocular abnormalities that affect the central visual field in the eyes with intermediate AMD, including glaucoma, diabetic retinopathy, and branch retinal vein occlusion.

### Diagnosis of hyperreflective foci on SD-OCT

At initial appearance, the presence or absence of an HRF was evaluated using SD-OCT. OCT scans were performed to cover the macular region using 25 horizontal B-scans spanning 20° × 25°. HRF was diagnosed according to previously published criteria^18^. Briefly, HRF was evaluated as well-defined and not confluent hyperreflective lesions located at any retinal layer in the macular area^19,20^.

The presence or absence of pseudodrusen was evaluated using multimodal imaging at initial presentation. The presence of pseudodrusen was defined when pseudodrusen was detected in at least one imaging modality, as previously described^21^. Drusenoid pigment epithelial detachment (DPED) was defined as an aggregated or confluent soft drusen > 350 µm in diameter on SD-OCT, as previously reported^22^.

### Follow-up visit

Three monthly intravitreal injections of vascular endothelial growth factor (VEGF) inhibitors, including ranibizumab or aflibercept, were administered to the contralateral eyes with MNV. Thereafter, injections were administered based on the treatment regimen. At each follow-up visit, color fundus photography and SD-OCT examination were performed in both the eyes, as performed at the initial presentation. FA/ICGA was performed to confirm the MNV subtype when intraretinal/subretinal fluid was detected on SD-OCT in the eyes with intermediate AMD. Atrophy was defined as cRORA on SD-OCT, as previously described^23^. The maximum follow-up duration was 60 months.

### Genotyping

At the initial appearance, peripheral blood of the participants was collected. Genomic DNA was obtained from peripheral blood using Pure Gene Isolation Kit (Gentra Systems, Minneapolis, MN, US). Genotype analyses were performed for *ARMS2* A69S and *CFH* I62V variants. Genotyping was performed using TaqMan assays on the ABI7300/7500 Real-Time PCR System (Applied Biosystems, Foster City, US), as previously described^24^.

### Statistical analyses

Statistical analyses were performed using Statflex 7 software (Artec Co., Ltd., Osaka, Japan). The differences in categorical and continuous variables between the two groups were analyzed using the chi-square test and Mann–Whitney U test, respectively. Cox regression analyses were performed to investigate the factors associated with progression to advanced AMD. Kaplan–Meier survival analyses were also performed to evaluate the influence of HRF on progression to advanced AMD. A *p* < 0.05 was considered to be statistically significant.

## Data Availability

The datasets analyzed during the current study are available from the corresponding author on reasonable request.
